# Cellular transcriptional profiling in human lung epithelial cells infected by different subtypes of influenza A viruses reveals an overall down-regulation of the host p53 pathway

**DOI:** 10.1186/1743-422X-8-285

**Published:** 2011-06-08

**Authors:** Olivier Terrier, Laurence Josset, Julien Textoris, Virginie Marcel, Gaëlle Cartet, Olivier Ferraris, Catherine N'Guyen, Bruno Lina, Jean-Jacques Diaz, Jean-Christophe Bourdon, Manuel Rosa-Calatrava

**Affiliations:** 1 Laboratoire de Virologie et Pathologie Humaine VirPath, Université Claude Bernard Lyon 1, Université de Lyon, Lyon, France; 2 Division of Medical Sciences, Centre for Oncology and Molecular Medicine, University of Dundee, Ninewells Hospital, Dundee, Scotland, UK; 3 Laboratoire de Virologie Centre de Biologie et de Pathologie Est, Hospices Civils de Lyon, Lyon, France; 4 Institut National de la Santé et de la Recherche Médicale (INSERM) U928 Technologies Avancées pour le Génome et la Clinique, Université de la Méditerranée, Marseille, France; 5 Centre National de la Recherche Scientifique (CNRS) UMR 5534, Centre Léon Bérard, Centre de Génétique Moléculaire et Cellulaire, Université Lyon 1, Lyon, France

## Abstract

**Background:**

Influenza viruses can modulate and hijack several cellular signalling pathways to efficiently support their replication. We recently investigated and compared the cellular gene expression profiles of human lung A549 cells infected by five different subtypes of human and avian influenza viruses (Josset *et al. *Plos One 2010). Using these transcriptomic data, we have focused our analysis on the modulation of the p53 pathway in response to influenza infection.

**Results:**

Our results were supported by both RT-qPCR and western blot analyses and reveal multiple alterations of the p53 pathway during infection. A down-regulation of mRNA expression was observed for the main regulators of p53 protein stability during infection by the complete set of viruses tested, and a significant decrease in p53 mRNA expression was also observed in H5N1 infected cells. In addition, several p53 target genes were also down-regulated by these influenza viruses and the expression of their product reduced.

**Conclusions:**

Our data reveal that influenza viruses cause an overall down-regulation of the host p53 pathway and highlight this pathway and p53 protein itself as important viral targets in the altering of apoptotic processes and in cell-cycle regulation.

## Background

Influenza viruses belong to the *Orthomyxoviridae *family of enveloped viruses containing a segmented genome of single stranded negative RNA. Among the three influenza types (A, B and C), type A is the most virulent pathogen with a diversity represented by the combination of 16 H and 9 N different subtypes (e.g. H1N1, H5N1) [1]. Influenza A viruses are the most serious threat to public health, with the potential to cause global pandemics as was illustrated in 2009 with the emergence of H1N1 SOIV [2].

All known subtypes of the influenza A virus are maintained in wild waterfowl, the natural reservoir of these viruses [3]. Current human circulating influenza A subtypes are H1N1 and H3N2. While extensive viral diversity is responsible for the subtype-specific virus-host interactions, many common functional features are also shared among viruses. Influenza infection alters host cellular homeostasis via the combination of the virally-induced alteration of biological machineries/pathways and the cellular antiviral response triggered by intracellular signalling cascades. Influenza viruses are able to activate/inhibit and hijack several cellular signalling pathways to efficiently support their own replication [4].

The development of high-throughput 'omic' studies has increased our understanding of viral-host interactions. Numerous studies have described host gene expression modifications induced upon viral infection *in vitro*, in animal models and in patients [5-9]. For example, a global mRNA profiling study of MDCK-infected cells has revealed an important role of the NF-kappaB signalling pathway for the H5N1 subtype and to a lesser extent for the currently circulating H3N2 strain [10]. Other intracellular signalling cascades are also induced by infection, in particular the mitogen-activated protein kinase (MAPK) and the PI3K/Akt pathways, both of which activate downstream transcription factors thereby affecting host gene expression [11,12]. While a small number of transcriptional studies have compared the cellular response induced by infection with highly pathogenic strains such as H1N1 1918 and H5N1 to that induced with low pathogenic strains, a systematic analysis and comparison of host cell mRNA expression during infection by several genetically diverging influenza subtypes has not yet been performed [7,8,13,14].

Using a nylon microarray, we recently characterized common gene expression changes induced during the infection of human lung epithelial cells by five different influenza A viruses. This shared signature was exploited to find new molecules that act on host metabolic pathways to bring about an antiviral effect against several subtypes [15]. Moreover, our systematic transcriptomic study also provided a gene expression database with which we compared the cellular responses induced by different influenza viruses. In the present study, we used the same data set to analyze strain specificity in regard to several host pathways. We observed that each viral signature was mainly associated with cellular pathways linked to cell death and cell cycle progression (*i.e. *cell growth). At the crossroads of all of these cellular processes, the p53 pathway became the focus of our investigation. Our analyses, validated by both RT-qPCR and western blot analyses, reveal multiple alterations within the p53 pathway during influenza A infection.

## Methods

### Cell lines, viruses and infection

Human lung epithelial A549 cells (ATCC CCL-185) or human colon carcinoma HCT116 cell lines were grown as monolayers in Dulbecco's modified Eagle's medium (DMEM, Gibco), supplemented with 10% heat-inactivated foetal bovine serum, 2 mM L-glutamine, 100 U/mL of penicillin and 100 mg/mL of streptomycin sulphate, at 37°C. The HCT116 cell lines containing a p53 wild-type (p53+/+) and a p53-deleted derivative (p53-/-) were gifts from Dr. Bert Vogelstein (John Hopkins University, Baltimore, MD, USA) [16]. Influenza viruses A/New Caledonia/20/99 (H1N1), A/Moscow/10/99 (H3N2), A/Lyon/969/09 (H1N1 SOIV), A/Turkey/582/2006 (H5N1), A/Finch/England/2051/94 (H5N2), and A/Chicken/Italy/2076/99 (H7N1) were produced in MDCK cells in EMEM supplemented with 2 mM L-glutamine, 100 U/mL of penicillin, 100 mg/mL of streptomycin sulphate and 1 mg/mL of trypsin. Viruses were titrated to determine the 50% tissue culture infective dose (TCID50) in MDCK cells as described in our previous study [17]. Confluent A549 cells were infected with influenza viruses at a MOI of 1 or 10-3 for one hour in a minimal volume of DMEM supplemented with 2 mM L-glutamine, 100 U of penicillin/mL, 100 μg of streptomycin sulphate/mL and 0.5 μg of trypsin/mL (infection medium) at 37°C. The cells were then overlaid with fresh infection medium and incubated at 37°C for 24 h. Viral kinetics for the six different influenza viruses have been determined previously [15]. No markers of apoptosis were detected at 24h post infection (MOI 1) by western blot (capspase 3 cleavage) and no signs of cell death were observed (data not shown).

### Microarray analysis

Gene expression data of A549 cells infected by H1N1, H3N2, H5N1, H5N2 or H7N1 influenza viruses or mock infected (five replicates in each group) were obtained in our previous study [15]. Data sets are available publicly from the Gene Expression Omnibus (GEO) database (http://www.ncbi.nlm.nih.gov/geo/) under accession number GSE22319. Five supervised analyses between groups of infected *versus *mock samples were conducted using the Significance Analysis of Microarray algorithm (SAM) [18], and the siggenes library (v1.18.0) [19]. Ingenuity Pathway Analysis 5.0 (IPA) (Ingenuity Systems, Redwood City, CA, USA) was used to select, annotate, and visualize genes according to function and pathway (Gene Ontology). Additional gene annotation was provided by the Interferome Database [20] and DAVID database (http://david.abcc.ncifcrf.gov/). Heat maps were produced by the heat map function from R that uses hierarchical clustering with Euclidean distance metric and the complete linkage method to generate the hierarchical tree (http://www.r-project.org/).

### Validation by RT-qPCR

Total RNAs were extracted using the RNAeasy Mini Kit (Qiagen). Reverse-transcription was performed on 1 μg of total RNAs using the Superscript II enzyme (Invitrogen) at 42°C. Quantification of the level of different mRNAs of interest was performed by real-time PCR on a MX3005P apparatus (Stratagene). Briefly, 20 ng of cDNAs were amplified using 0.8 μM of each primer, 0.4 μM of probe (cf. Table 1) and 1X Taqman Universal Master Mix (Applied Biosystems). All data were normalized to the internal standard Actin. For each single-well amplification reaction, a threshold cycle (Ct) was observed in the exponential phase of amplification. Relative changes in gene expression were determined using the 2ΔΔCt method and reported as the n-fold difference relative to a control cDNA (mock, uninfected cells) prepared in parallel with the experimental cDNAs (infected cells) [21]. The mRNA levels were measured in triplicate in two independent experiments. Quantification of M viral genome copies released in supernatants was performed according to previously published work [22].

**Table 1:** List of primers used in this study

Primers & probes		Sequence 5'-3'
**p53 (Exon8-9)**	Forward	GAA GAG AAT CTC CGC AAG AAA GG

	Reverse	TCC ATC CAG TGG TTT CTT CTT TG

	Probe	AGC ACT AAG CGA GCA CTG CCC AAC

**p21**	Forward	GACTCTCAGGGTCGAAAACGG

	Reverse	GCGGATTAGGGCTTCCTCTT

	Probe	CTACCACTCCAAACGCCGGCTGATCT

**Bax**	Forward	ACTCCCCCCGAGAGGTCTT

	Reverse	GCAAAGTAGAAAAGGGCGACAA

	Probe	GAGCTGACATGTTTTCTGACGGCAACTTCAACT

**Bcl-XL**	Forward	TCC TTG TCT AGG CTT TCC ACG

	Reverse	GGT CGC ATT CTC GCC TTT

	Probe	ACA GTG CCC CGC CGA AGG AGA

**Influenza M**	Forward	CTTCTAACCGAGGTCGAAACGTA

	Reverse	GGTGACAGGATTGGTCTTGTCTTTA

	Probe	TCAGGCCCCCTCAAAGCCGAG

### Western blot analysis

Total proteins were extracted by scraping and syringing cells in 1X NuPAGE LDS buffer (Invitrogen). Fifteen to thirteen micrograms of total proteins were then separated on pre-cast 10% NuPAGE gels (Invitrogen). To detect the different proteins of interest, the following antibodies were used: mouse monoclonal DO-1 (p53) and SX118 (p21, Cell signalling), Rabbit polyclonals Bax, Akt and Bcl-XL (Cell signaling). The mouse monoclonal Ku80 antibody was used as a loading control (Abcam). The relative protein levels (RPL) were calculated by densitometry analysis performed with the help of the ImageJ software (http://rsbweb.nih.gov/ij/).

## Results

### Specific viral signatures and commonly targeted host genes

**Figure 1:**
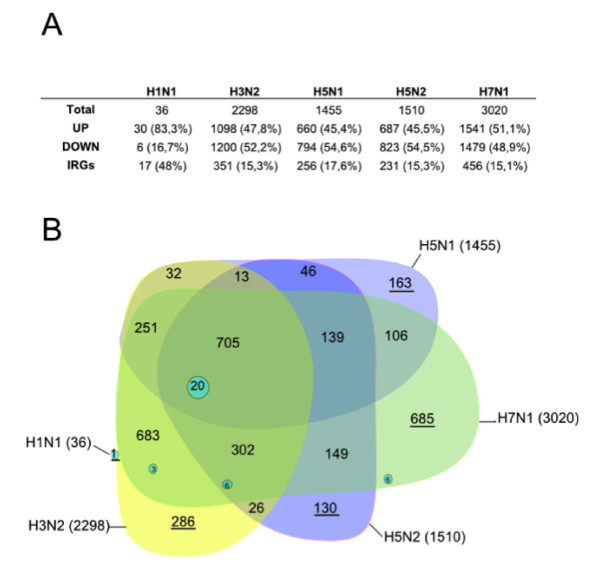
**Summary of genes expressed in response to H1N1, H3N2, H5N1, H5N2 and H7N1 viral infection**. **A**. Genes significantly regulated (SAM, FDR = 10%) in response to the different influenza viruses compared to mock infection at 24 hpi are shown. **B**. Venn-diagram showing the genes co-regulated by several viruses

We have previously reported variations in gene expression at 24 h post-infection in human pulmonary epithelial A549 cells infected with different subtypes of influenza viruses: human A/New Caledonia/20/99 (H1N1), A/Moscow/10/99 (H3N2), avian A/Turkey/582/2006 (H5N1), A/Finch/England/2051/94 (H5N2), A/Chicken/Italy/2076/99 (H7N1) influenza viral strains or mock control infection (five replicates in each group) [15]. Compared to the mock control infection, changes were observed in 36 cellular genes for H1N1, 2298 genes for H3N2, 1455 genes for H5N1, 1510 genes for H5N2 and 3020 genes for H7N1 (SAM algorithm with a False Discovery Rate of 10%). The small changes induced upon H1N1 infection were correlated to its low level of replication in our experimental conditions [15]. While 83% of the host genes altered following H1N1 infection were over-expressed, a similar number of genes were over- and under-expressed for each of the other viruses (figure 1A). Furthermore, we found that the expression levels for a total of 300 genes (representing 3.4% of the genes considered present on the chip) differed significantly between mock and all infected samples including H1N1 [15].

To determine the specific cellular genes showing altered expression within each of the different strain-specific signatures, we built the Venn diagram shown in figure 1B. Common changes in the expression of numerous genes (1007) were induced by three of the viruses, H3N2, H5N2 and H7N1. The expression of 705 of these genes was also altered when four viruses were considered: H3N2, H5N1, H5N2 and H7N1. Since H1N1 deregulated only a small number of genes, the number of those the expression of which was modified by all strains was limited to 20. Their associated biological responses were described in our previous study and are mainly linked to the immune response in accordance with other investigations [15].

### Biological processes associated with each viral signature

**Figure 2:**
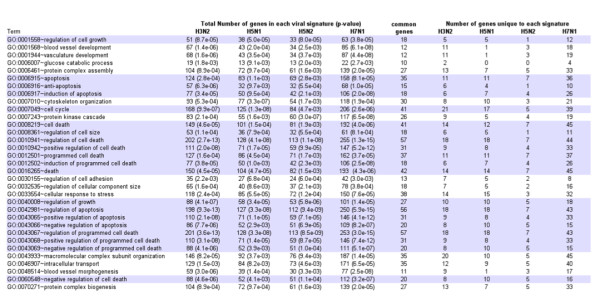
**Significantly enriched GO Biological Processes (p-value < 0.01) common to H3N2, H5N1, H5N2 and H7N1 specific signatures**. Terms related to the p53 pathway are highlighted in grey.

We then determined the Gene Ontology (GO) biological processes (or terms) significantly associated with each viral signature using the DAVID database. With a p-value < 0.01, no one term was shared among the five viruses. The 36 genes deregulated upon H1N1 infection encode proteins involved in the cellular immune response, transcription, biogenesis of protein complexes and nucleic acid metabolic processes. In contrast, the signatures of H3N2, H5N1, H5N2 and H7N1 viruses were each associated with 33 common terms (p-value < 0.01) (figure 2), covering all the biological processes of DAVID database. Two major classes of biological processes could be distinguished. Firstly, there are those that are affected by the four viral strains through the modulation of a common gene subset. For example, each of the viral strains modulated the expression of 10 common genes involved in the glucose catabolic process", while only a few genes involved in this process were differentially modulated in a strain-dependent manner. Secondly, there are those that are affected by each of the four viral strains through the modulation of distinct gene subsets in a strain-dependent manner. For example, while 35 genes all associated with the "apoptosis" process were modulated by the four viruses, 11 of these were specifically modified upon H3N2 infection, 11 by H5N1, 7 by H5N2 and 35 by H7N1.

Among the 33 common terms associated with the viral signatures, more than 60% (n = 20) were related to the control of cell death or cell cycle progression. Other common biological processes were related to angiogenesis, protein complex biogenesis or metabolism [15]. Interestingly, despite the abovementioned current association made between influenza virus-induced host cell responses and the cellular immune response, we found no terms shared by all viruses relating to the immune response. Moreover, by consulting the whole list of biological processes associated to each virus signature we found the term "immune response" significantly mapped to each viral profile, except for the H5N2 virus. However, the comparison of our differentially expressed gene lists to Interferome, an IFN-regulated gene database, revealed that a similar proportion of genes regulated upon infection by H3N2, H5N1, H5N2 or H7N1 (around 15%) was related to the IFN response, whereas 48% of the genes responding to H1N1 infection were regulated by IFN [20].

In summary, analysis of our data showed that more than half of the genes responding to infection by H3N2, H5N1, H5N2 and H7N1 viruses, are involved in cell death, cell cycle progression or the cellular immune response. All these biological processes are regulated by a central signalling pathway, the p53 pathway [23]. Indeed, in addition to regulating the expression of genes involved in apoptosis and cell cycle arrest, p53 also regulates the expression of type I IFN as well as several genes carrying IFN-stimulated response elements [24]. For these reasons, and in light of several proposals of p53 being a functional interactant of influenza [25-27], we focused our investigations on modifications of the p53 pathway in response to influenza infection.

### Infection induces a transcriptional down-regulation of the upstream signal part of the p53 pathway

In response to stress, such as viral infection, several signalling mediators relay the stress stimuli to the p53 protein, which is consequently stabilized and activated mainly by post-translational modifications including phosphorylations and acetylations [28]. Such modifications disrupt interactions between p53 and the ubiquitin ligase Hdm2, responsible for the rapid degradation of p53 in basal conditions [29,30]. Once activated, p53 regulates cell cycle arrest and apoptosis through the direct modulation of host cellular gene expression.

**Figure 3:**
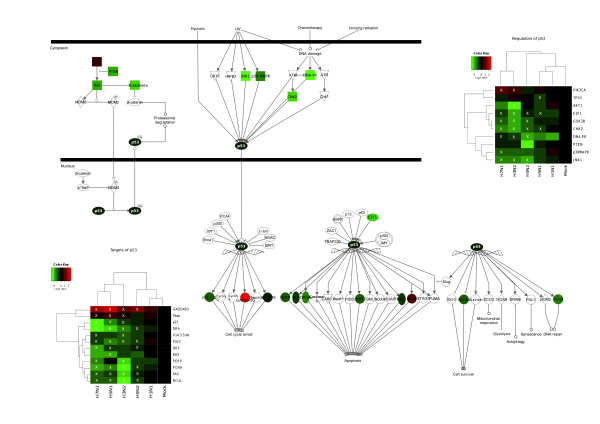
**Transcriptional regulation of the genes belonging to the p53 signalling pathway (IPA canonical pathway) during influenza viral infection**. Mean fold changes of the genes encoding regulators and targets of p53 that were differentially expressed by at least one virus, calculated as Log_2_(Mean expression levels in infected samples) - Log_2_(Mean expression levels in mock samples) and overlaid on the pathway in green for downregulated genes (FC < 0), and red for upregulated genes (FC > 0). Fold changes in each group of samples are depicted in the heat maps and were used to cluster hierarchically both samples and genes. Genes considered significant for each virus with the SAM procedure are indicated by a white X on the heat maps.

We firstly focused our attention on the upstream signal part of the p53 pathway. According to our data, all the main kinases known to directly regulate p53 phosphorylation status (DNA-PK, Chk2, JNK1 and Gsk3β) are under-expressed following infection by H3N2, H5N1, H5N2 and H7N1 viruses (figure 3). For example, n-fold *DNA-PK *mRNA changes of -1.78, -2.12, -1.41 and -1.56 were measured for H3N2, H5N1, H5N2 and H7N1, respectively (figure 3). We also focused our attention on the phosphatidylinositol 3-kinase (PI3K)/Akt pathway, which directly regulates Hdm2 expression and thus endogenous p53 activity [31]. Interestingly, our results indicate a decreased expression of both *AKT1 *and its negative regulator *PTEN *(figure 3). These results are in accordance with the activation of the phosphatidylinositol 3-kinase (PI3K)/Akt pathway during influenza infection, as already described in the literature [32].

Altogether, our results reveal that influenza A infection induces the transcriptional down-regulation of several genes encoding host factors belonging to the upstream signal part of the p53 pathway and which act on the endogenous activity of p53.

### Differential effects of influenza viruses on p53 mRNA and protein levels

**Figure 4:**
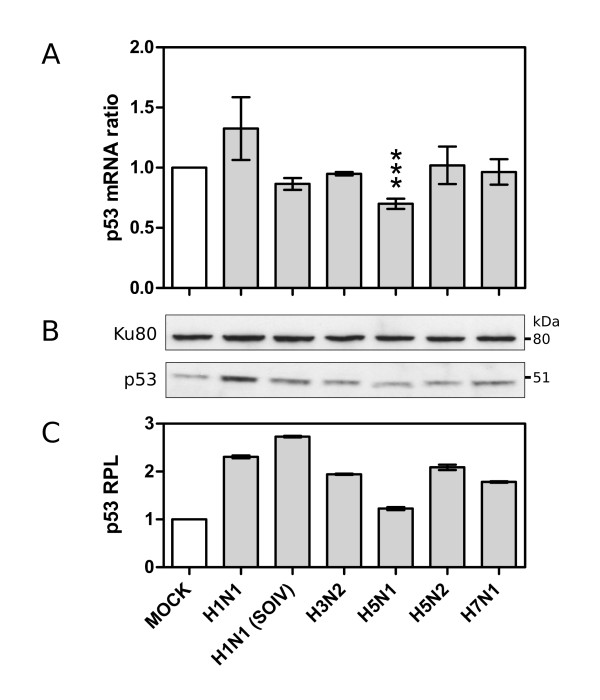
**A. Validation of microarray data by RT-qPCR**. The measured ratio change of p53 mRNA levels was subject to statistical analysis (student t-test, *** p-value < 0.001). **B**. p53 protein levels revealed by western blot. **C**. Relative protein levels (RPL) measured by densitometry analysis.

Secondly, we assessed whether viral infection could also directly affect p53 mRNA expression. Interestingly, our data indicates that only one of the investigated viruses, H5N1, led to a significant decrease in the level of p53 mRNA expression, with a fold change of -1.54 (figure 3). To assess such differential data, we infected another set of A549 cells with the different viruses used in our initial microarray study, including the low replicative H1N1 virus to which we added the recent pandemic H1N1 SOIV as an efficient productive H1N1 virus [28]. Total cell RNA was extracted 24 hours post-infection, similarly to that performed for the microarray analysis. The quantification of p53 mRNA levels was carried out by RT-qPCR using primers matching several different EST sequences used in the microarray chip. In accordance with our previous data, no significant change in the p53 mRNA ratio was detected with H3N2, H5N2 and H7N1, nor with H1N1 or H1N1 SOIV, while a significant ratio change of 0.70 was observed in H5N1 infected cells (p < 0.0005, figure 4A). The calculated fold change was -1.43, indicating the specific down-regulation of p53 mRNA expression in the context of H5N1 infection.

We further investigated whether such influenza-induced variations in p53 mRNA levels could be correlated or not with changes in the endogenous p53 protein level. For that, we reproduced the same experimental conditions as described above and performed western blots on infected cellular extracts. Compared to mock-infected cells, the p53 protein levels (Relative Protein Level, RPL) measured by densitometry analysis were significantly increased in cells infected by all the viruses studied, with a 1.78 to 2.72 fold increase for respectively H7N1 and H1N1 (SOIV), and a smaller 1.22 fold increase for H5N1 (figure 4B and 4C).

Our data further support the previously reported non-correlation between p53 mRNA and protein levels in response to influenza infection, and are also in line with several other investigations reporting such a mismatch in the context of other cellular stresses [33]. Moreover, our results reveal an interesting contrast between H5N1 and the other viruses. Indeed, H5N1 infection directly affects the p53 mRNA expression level, accounting for the attenuated increase in the p53 protein level observed in the H5N1 infected cell.

### Transcriptional down-regulation of downstream signalling of the p53 pathway upon infection

Having determined an increase in p53 protein levels during the time-course of infection, we then focused our attention on the expression of p53 downstream target genes. While several studies have previously shown an increase in p53 transcriptional activity during influenza virus infection by luciferase assay or the detection of phosphorylated p53 in infected cells [25,34], we observed in our experimental conditions a significant decrease in the transcriptional expression of several p53-targets genes during infection by H3N2, H5N1, H5N2 and H7N1 viruses (figure 3). These down-regulated genes include *p21*, *14-3-3*, *PERP*, *FAS*, *DR4/5*, *PIG3*, *BAX*, *Bcl-XL*, *PAI-1 *and *PCNA. *For p21 (*CDKN1A) *for example, the n-fold mRNA changes measured were -1.81, -2.0 and -1.85 for H3N2, H5N1 and H7N1 infections, respectively. The expression of only two p53-target genes investigated, *Teap *(*TP53INP1*), and *GADD45G *was increased upon infection by the 4 viruses (figure 3). The *GADD45G *n-fold mRNA changes measured were 1.61, 1.70, 1.26 and 1.60 for H3N2, H5N1, H5N2 and H7N1, respectively. It should be noted that *TP53INP1 *is also known to be regulated by p73, a p53 homologous protein, and must therefore be also regulated in a p53-independent manner [35].

**Figure 5:**
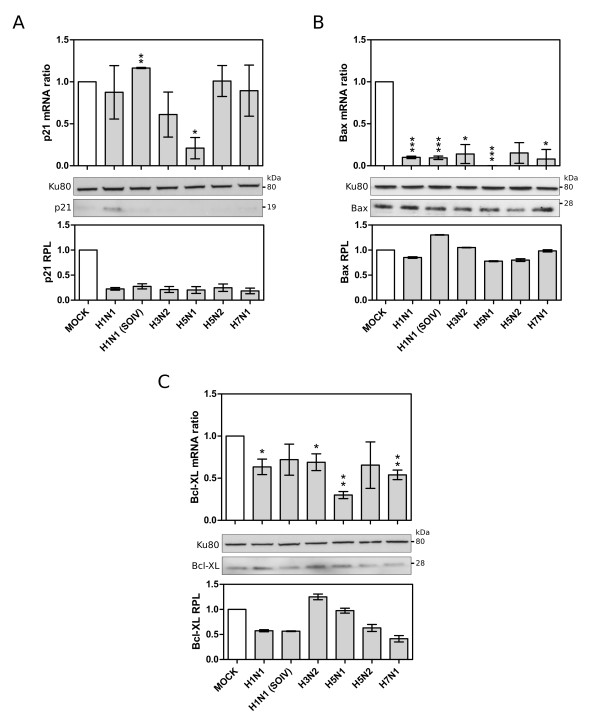
**Validation of microarray data by RT-qPCR and western blot: p53 target genes, (A) p21, (B) Bax, (C)Bcl-XL**. (*, ** and *** correspond to p-values <0.05, <0,005, <0.001, respectively).

To support these results, another set of A549 cells were infected with the broad set of viruses for 24 hpi and variations of endogenous p21, Bax and Bcl-XL mRNA expressions and protein levels were analyzed in parallel by RT-qPCR and western blot, respectively (figure 5A, B and 5C). For p21, the mRNA level was significantly reduced only in H5N1-infected samples (p < 0.05, mRNA ratio around 0.2), while the protein levels were reduced in cells infected by almost all the viruses studied (figure 5A). Moreover, a down-regulation of *Bax *expression was also clearly observed with significantly reduced mRNA levels in all infected cells, despite only minor changes at the protein level (figure 5B). We also confirmed the down-regulation of *Bcl-XL *expression in cells infected by all viruses except H1N1 SOIV and H5N2 (figure 5C). At the protein level, Bcl-XL protein level was decreased by most viruses. Of note, the down-regulation of the three p53-target genes, *p21*, *BAX *and *Bcl-XL*, was considerably greater in the context of H5N1 infection than with the other influenza A viruses.

Altogether, our results reveal that influenza A infection leads to the down-regulation of several p53 transcriptional targets, at both the mRNA and protein levels for some, despite the relative increase in p53 protein levels common for all viruses.

### p53 contributes to an antiviral cellular state

**Figure 6:**
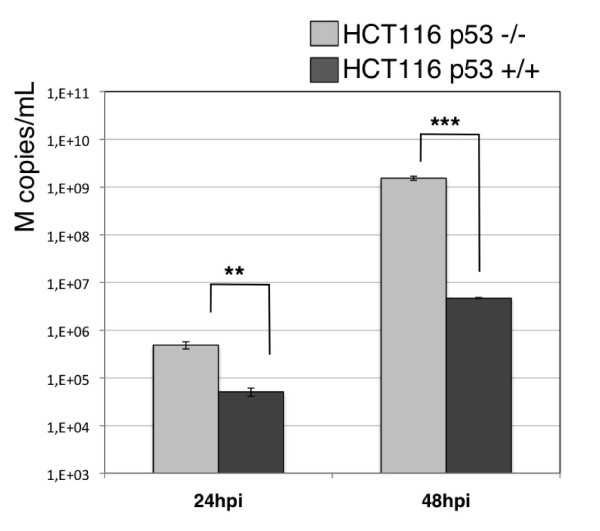
**Comparison of viral titres (copies M/mL) produced in HCT116 p53 +/+ or -/- infected with H3N2 A/Moscow/10/99, calculated by RT-qPCR of M genome copies released in the supernatants**.

We then wished to assess whether such a global down-regulation of the p53 pathway could be associated with a functional effect with regards to viral replication. For this, we compared the production yield of one representative influenza virus (subtype H3N2, circulating strain A/Moscow/10/99) in the HCT116 isogenic cell models (figure 6). HCT116 p53+/+ and p53-/- cells were infected with H3N2 at a MOI of 10-3 and the released viral genome copies were extracted from culture supernatants and quantified by RTqPCR at 24 and 48 hpi, as described in materials and methods. The number of genome copies reflecting viral production was 10 and 100 fold higher in HCT116 p53-/- than in HCT116 p53+/+ cells at 24 and 48h post-infection, respectively. These results show the effectiveness of p53 protein at slowing down viral production. In agreement with previous reports [6,25], these data suggest the contribution of the p53 protein and associated pathways to the general cellular antiviral response against influenza infection and support the hypothesis that the virally induced global down-regulation of the p53 pathway leads to a cellular state which favours their replication.

## Discussion

### A pathway of interest to study influenza interactions with the host cell

In this present study, we report a microarray analysis revealing that infection by four human or avian influenza viruses (H3N2, H5N1, H5N2 and H7N1) significantly alters the gene expression of several host factors belonging to the p53 pathway. This pathway is composed of numerous genes, the protein products of which have a direct impact on host cell homeostasis, via the regulation of cell death and cell cycle progression as well as several other processes such as metabolism or the cellular immune response [23]. The transcriptional activity of p53 is tightly regulated by Hdm2 in response to several intracellular signalling cascades triggered by different stimuli (DNA damage, UV irradiation and hypoxia). The central core of the pathway includes the p53 protein itself, which can directly or indirectly shut down the growth of stressed cells mainly by inducing cell-cycle arrest and apoptosis. Considering the central role of the p53 pathway, the impact of influenza infection on this cellular pathway is of particular interest.

Several studies have underlined the interplay between influenza viruses and different cellular signalling pathways, such as the PI3K/Akt, innate immune or apoptotic signalling pathways [4,36-39]. Intriguingly however, only a few have been dedicated to changes in p53 upon influenza infection, highlighting the need to further characterize the interplay between influenza viruses and the p53 pathway [25,26,34,40].

### Influenza infection is associated with a global down-regulation of signalling cascades both upstream and downstream of p53 protein

We first observed that upstream signals of the p53 pathway that directly connect stress (UV, hypoxia, DNA damage) -induced signalling cascades to regulators of p53, were generally down-regulated at the transcriptional level by infection. Among such regulating kinases, *DNA-PK*, *Chk2*, *JNK1 *and *Gsk3β *genes are all under-expressed following infection.

Similarly, our results also revealed a significant decrease in gene expression for several endogenous p53 transcriptional targets, including *p21*, *14-3-3*, *PERP*, *FAS*, *DR4/5*, *PIG3*, *BAX*, *Bcl-XL*, *PAI-1 *and *PCNA *during infection by our set of viruses. Moreover, these results were confirmed at the protein level for p21, Bax and Bcl-XL.

Altogether, our results indicate a global down-regulation in the mRNA expression of principal factors belonging to both upstream and downstream parts of the p53 pathway, suggesting a global negative effect on p53 pathway activity.

### Discrepancy between p53 mRNA, protein levels and activity in influenza infected cells

The down-regulation of p53-target genes suggests that p53 expression and consequently its transcriptional activity is decreased during infection. We verified whether this was the case using luciferase assay and the monitoring of p53 phosphorylation status by western blot in H3N2-infected A549 cells (data not shown) and revealed some discrepancies. On the one hand, no significant change in p53 mRNA expression was detected in infected cells, except for a significant decrease with H5N1. On the other hand, levels of p53 protein expression were significantly increased by all the viruses, seemingly incoherent with the reduced activity of p53 in infected cells.

Altogether, these results suggest a likely complex regulation of *TP53 *expression during infection. Regulating factors include transcriptional activators (ISGF3 PRKCδ, HOXA5, CREBPB), inhibitors (BCL6, RBPJ) and stabilizing factors (ELAVL1 and ZMAT3) [33]. Influenza infection might therefore induce the over-expression of both inhibitors (*BLC6 *and *RBPJ) *and activators (*ISGF3) (data not shown)*. In accordance with this notion, a recent study reported the stress-induced regulation of p53 activity through the control of p53 mRNA stabilization and translation in addition to the well-described alteration of protein stability [33]. Other stabilizing/destabilizing factors, such as host microRNAs may also be involved and should be investigated in the context of infection [33], in particular with H5N1, which induces a down-regulation of p53 mRNA expression unlike the other viruses.

Our results also clearly reveal that the impact that influenza viruses have on p53 is not exclusively on gene expression. Further investigations are necessary, in particular on the stabilization and activation of p53 protein, considering the putative interaction of p53 with viral proteins during the time-course of infection.

In this perspective, a recent study reported that the viral protein NS1 interacts with p53 and inhibits its activity [27]. Furthermore, transient expression of H5N1 NS1 (wild-type or with mutations in regions 80-84 and 92) reduced the transcriptional activity of p53 [41]. We can hypothesize that p53 protein is stabilized upon infection in an inactive form through interaction with the viral NS1 protein [27]. Such a scenario would explain our seemingly contradicting results concerning p53 expression and activity. This may even reflect what happens with a number of DNA viruses for which viral proteins like BZLF1 (Epstein Barr Virus) or HBx (Hepatitis B virus) inhibit the transcriptional activity of a stabilized form of p53 [42,43]. Interestingly, the disruption of p53 signalling to p21 was described in human lung A549 cells in the case of BZLF1 viral protein [44].

### Viral down-regulation of the p53 pathway and modulation of the cell cycle and apoptosis by influenza A infection

We report the down-regulation of the p53-target genes *p21 *(G1/S-arrest) and *14-3-3 *(G2/M-arrest by sequestering Cdc2-complex Cyclin B), both of which are involved in cell cycle control. As expected, the down-regulation of *p21 *and *14-3-3 *in association with an over-expression of the cell-cycle regulators *TP53INP1 and GADD45G *[35,45], could modulate cell cycle progression and resulted in G1-arrest. Our results are in line with one recent study describing the effect of influenza A infection on the cell cycle and show that viruses can induce an arrest in G0/G1 [46]. They also suggest a putative involvement of p21, TP53INP1 and Gadd45gamma in the influenza-induced cell cycle arrest. A thorough functional evaluation of these results is now needed which should contribute to a better understanding of the viral mechanisms involved which permit an optimal viral protein expression and progeny production.

The other group of down-regulated p53-target genes that we identified during infection encodes the pro-apoptotic proteins PERP [47], Fas, DR4/5, PIG3 and BAX. In addition, the Bax-inhibiting[48] anti-apoptotic factor Bcl-XL, was also under-expressed during infection, as was PAI-1 whose role in regulating apoptosis remains unclear [49]. At the protein level, Bcl-XL expression was decreased by most viral infections. It is worth noting that the down-regulation of the three p53-target genes, *p21*, *BAX *and *Bcl-XL*, was considerably greater after H5N1 infection than with the other influenza A viruses tested.

Concordantly, the knock-down of *BAX *was recently shown to reduce the replication of influenza virus [50]. Thus the down-regulation of a negative regulator of Bax, such as the anti-apoptotic Bcl-XL, at both mRNA and protein levels, such as we have reported here, would be expected to increase viral replication. Further silencing experiments are required to confirm such a result.

Altogether, our observations suggest that influenza viruses may at least partially control intrinsic and extrinsic pathways of apoptosis by decreasing the expression of both pro- and anti-apoptotic factors which are under the control of the p53 transcription factor, as suggested by several studies [39].

### Is inhibition of the p53 pathway essential for the replication cycle of influenza viruses?

In agreement with the above hypothesis, we showed that the level of H3N2 viral production is significantly higher in HCT116 p53 -/- than in HCT116 p53 +/+ cells, at both 24 and 48 hpi (figure 6). These results are in accordance with initial observations reported by Turpin and colleagues in a p53 dominant-negative A549 model and more recent data reported on siRNA high-throughput screening for host functional interactants [25,6].

These and our results suggest a marked antiviral facet for p53 and its pathway in the context of influenza infection, as has already been observed for several other viruses. The antiviral effect of p53 depends largely on its pro-apoptotic activity, which limits virus replication [51]. However, other works have also demonstrated that p53 contributes to the innate cellular antiviral response by enhancing type I interferon (IFN)-dependent antiviral activity, independently of its function as a pro-apoptotic gene [52]. In light of our results on p53 targets implicated in cell-cycle regulation and a recent study on human circulating leukocytes from infected patients [53], the p53 antiviral role might involve not only its activity in mediating the IFN response and apoptosis, but also in controlling cell cycle progression.

## Conclusions

In conclusion, the p53 pathway is inhibited during infection by influenza A, either by the control over the expression of key p53 stabilizing factors, as is the case with H3N2, H5N1, H5N2 and H7N1, or by the decrease in p53 mRNA levels as is the case with H5N1. In accordance with an overall decrease of p53 activity, the global down-regulation of p53-target genes could contribute to changes in both the apoptotic response and cell-cycle progression, altogether inducing a pro-viral cellular context. The present study has highlighted the p53 pathway as an important player in influenza infection. Further investigations are now needed to discriminate the cellular antiviral response from the viral hijacking of the p53 pathway during the time-course of infection.

## Competing interests

The authors declare that they have no competing interests.

## Authors' contributions

TO and LJ carried out the experiments and the analysis of results. TO, LJ and MRC wrote the manuscript. GC and OF participated in the design of the study and performed viral infections in the human lung carcinoma A549 cellular model. JT supervised the transcriptomic analysis. CN, JJD, BL, VM and JCB participated to the conception and coordination of the study and helped to write the manuscript. MRC managed the investigations. All authors read and approved the final manuscript.

## Fundings

This work was supported by University Claude Bernard of Lyon, the Civil hospitals of Lyon, the AVIESAN alliance and by a grant of the "Programme de Recherche A(H1N1)" co-ordinated by the Institut de Microbiologie et Maladies Infectieuses (INSERM). The funders had no role in study design, data collection and analysis, decision to publish, or preparation of the manuscript.
